# Hierarchical drivers of soil microbial community structure variability in “Monte Perdido” Massif (Central Pyrenees)

**DOI:** 10.1038/s41598-019-45372-z

**Published:** 2019-06-19

**Authors:** Juan J. Jiménez, José M. Igual, Luis Villar, José L. Benito-Alonso, Jesús Abadias-Ullod

**Affiliations:** 1 Department of Biodiversity and Ecosystem Restoration, Pyrenean Institute of Ecology (IPE), Spanish National Research Council (CSIC), Jaca (Huesca), Spain; 2 Instituto de Recursos Naturales y Agrobiología de Salamanca (IRNASA-CSIC), Salamanca, Spain; 3 Jolube consulting (www.jolube.es), Mariano Rodríguez de Ledesma, 4, 22700 Jaca, Spain

**Keywords:** Microbial ecology, Ecosystem ecology

## Abstract

Microbial activity is highly dependent on climatic factors (moisture and temperature) and edaphic characteristics in temperate ecosystems. Moreover, soil microbial community composition in high mountain areas is less known when compared to plant communities. In this study we investigated the soil microbial community from a functional perspective using PLFA (phospholipid fatty acid) methods in the four aspects of four summits (2,242 – 3,012 m above sea level) in the Spanish Central Pyrenees. Soil organic carbon (C), microbial biomass and nutrient dynamics ($N{H}_{4}^{+}$ + $N{O}_{3}^{-}$, N mineralization and nitrification potential) were also determined. Microbial biomass C was highest in the lowermost summit and decreased by approximately 50, 14 and 12% with increasing altitude. In each summit soil $N{H}_{4}^{+}$ and $N{O}_{3}^{-}$ concentrations differed significantly among summits and aspects. Soil nitrification potential varied significantly between the factors summit and aspects, e.g., southerly vs. northerly, easterly vs. westerly aspects. Gram negative bacteria and Actinobacteria functional groups dominated the microbial community, with almost 40% of the total PLFA. Non-metric multidimensional scale (NMS) analysis showed that most of the PLFA functional groups were present in all summits and aspects, although with specific biomarkers. A high abundance of biomarkers 16:1ω9c and 16:0 2OH (gram negative bacteria) were obtained in the lowermost summit, while the biomarkers 16.1ω7cDMA (anaerobes) and 19:3ω6c (Eukaryote) were only found in the uppermost summit. Linear mixed model (lmm) analysis was used with summit as fixed effect and aspect as random effect. In general, our results demonstrate a fundamental role for environment, principally moisture, temperature and organic matter in explaining the pattern observed for soil PLFA biomarkers. Under a global change scenario, we need to shed light on the relationships between soil microbial functional groups and soil nutrient-related variables in order to identify the associated patterns of decomposition rates and soil processes driven by microbial communities in mountain areas. The results could thus be used in global predictive models on climate change impact on C or N cycles in these environments.

## Introduction

Soil microorganisms are critical to C and N fluxes in terrestrial ecosystems^1,2^, as they thrive important processes related to nutrient cycling like mineralization of soil N for plant uptake. They are also highly dependent on climatic factors (moisture and temperature), edaphic characteristics such as clay mineralogy, and the physical and chemical characteristics of the decomposing resources^3^. Mountain ecosystems are fragile environments that play a role as early warning systems due to their sensitivity to global warming^4^. It is thus expected that soil functions like N mineralization and nitrification in these areas will be highly affected by global change (GC)^5^. Related GC factors like nitrogen deposition, elevated carbon dioxide (CO_2_) concentrations in the atmosphere, wind and water erosion, land use changes, and altered disturbance regimes are expected to affect plant community composition and cover, net primary productivity (NPP) and carbon (C) storage^6,7^. They will also affect soil decomposition rates by changing the composition and community structure of soil microorganism. Some studies have reported the effect of plant cover on microbial communities and/or their functions^8^ or on plant-soil interaction under GC context^9–12^, along altitudinal gradients, including arctic-alpine ecosystems^13–18^.

The ongoing reduction of snow cover in Alpine ecosystems by increasing temperatures due to global warming is predicted to affect plant dynamics and vegetation cover with progressive decline of cold mountain habitats and biota and a reshuffling of species on altitude gradients is to be expected as a consequence of individualistic species responses^19^. A warming effect called thermophilization, the process by which cold adapted plant species are progressively replaced by warm adapted species, has been described across a range of European mountainous areas^19,20^. The aspect of a slope has also been identified as a principal determinant of the pace of climate-induced plant migration processes^21^. In temperate regions of Europe, east- and south facing mountain slopes favour site-specific plant species richness, compared to the western and northern sides of the same mountains. Even at the highest altitudes plant productivity can be higher in the southern aspects compared to other aspects at same altitudes as a result of less cold temperatures, with shorter periods of snow cover and higher sunshine^4^. The upward migration of plants as a response to CC can be associated with concomitant changes in soil processes, such as decomposition, as an altered temperature regime can affect the turnover of soil nutrients and plant community structure. Consequently, the general reduction of plant productivity with increasing altitude occurring in mountain areas^22^ is expected to be counteracted in southern aspects through lower climatic constrains on organic matter (OM) decomposition.

In mountainous areas, abiotic factors such as erosive forces, thaw-freezing cycles prevail at high altitudes, while more anthropic driven factors like land husbandry are strong determinants of soil nutrient-related processes at lower altitudes. Nutrient-related processes in mountain soils with changes related to the forecasted temperature increase, rainfall pattern variations and plant cover dynamics in the next decades^4^ must be addressed.

The abundance of decomposer soil fungi, bacteria and actinobacteria is primarily driven by plant production, and hence mostly responsive to the quantity of plant inputs to soil^23^. Although some studies have documented the relationship among microbial biomass, microbial activity and N transformations in mountain summits^24,25^, in our research site no study has been done so far that relates microbial composition with soil environmental factors. Thus, more studies are needed to be used to inform predictive models on e.g. climate change impact on soil C or N cycles in these environments.

Baseline data are thus needed to be compared with future assessments of soil nutrient dynamics and related microbial community composition in mountain ecosystems. By so doing increased understanding of microbial mechanisms involved in SOM decomposition under changing climate will be achieved^26–28^. In four summits of Spanish Central Pyrenees we tested the hypothesis that (i) specific PLFA biomarkers varied across summits, (ii) available soil nutrient concentrations decreased with altitude and, (iii) microbial community composition and ecosystem processes like decomposition was influenced by a set of environmental factors across the accepted classical hierarchy of drivers^8^. Thus, the aim of this study was (i) to characterize the microbial community PLFA composition and its relation with soil N availability, N mineralization and nitrification potential.

## Results

### Soil properties

Total C, N and P decreased with altitude while pH increased (Table 1). The highest concentrations of total N were obtained in the two lower summits, while the lowest values were found in the two uppermost summits. Total P concentration was lowest in the two uppermost summits, i.e., less than 0.1 ppm. In the two lower summits, the lowest P concentration was found in ACU east and CUS north, while the highest values were found in ACU north and CUS west (Table 1). Soil pH was lowest in ACU west and highest in CUS north and OLA East. The analysis of soil elements showed that Ca^2+^, Mg^2+^ and K^+^ concentrations were highest at CUS west, while Na^+^ concentration was lowest. In general cation concentrations were lowest in the uppermost summit, OLA (Table 1).

**Table 1:** Main soil physico-chemical characteristics (0–5 cm) in the four summits analysed.

Summit	Aspect	Texture	Sand	Silt	Clay	pH	OM^†^	SOC	Total N^††^	Total P	C:N	N/P	MBC^¶^	MBN^¶¶^	Cmic:	Ca	Mg	Na	K
(m a.s.l.)			%	%	%	H_2_O (1:1)	%	g kg^−1^	%	ppm			<–µg g dry soil^−1^>	Corg	<--------------- ppm -------------->				
ACU (2,200)	N	Sandy loam	60.0	26.4	13.6	6.6	8.09	52.5	0.49	0.53	10.71	0.92	443.63	31.99	8.45	47.75	2.88	4.56	8.57
	S	Loam	34.5	42.0	23.5	5.9	4.33	27.28	0.32	0.385	8.53	0.85	683.49	38.99	25.05	61.02	14.70	7.27	151.58
	E	Loam	40.1	36.3	23.5	6.3	4.12	27.1	0.28	0.142	9.68	1.95	780.87	38.82	28.81	27.99	3.41	2.78	19.79
	W	Clay loam	31.6	40.7	27.7	4.0	9.25	66.21	0.54	0.224	12.26	2.44	1334.09	46.38	20.15	22.02	4.47	3.90	12.76
	**Mean**		**41.6**	**36.4**	**22.1**	**5.7**	**6.4**	**43.3**	**0.4**	**0.32**	**10.3**	**1.5**	**810.5**	**39.0**	**20.6**	**39.7**	**6.4**	**4.6**	**48.2**
CUS (2,550)	N	Silt loam	18.9	60.5	20.6	7.9	1.54	10.78	0.14	0.009	10.00	15.85	350.99	18.09	32.56	56.25	4.25	2.49	2.25
	S	Loam	45.1	36.3	18.6	6.5	7.10	52.45	0.40	0.69	13.11	0.58	816.20	16.39	15.56	116.02	14.14	9.15	77.09
	E	Silt loam	19.8	66.8	13.4	7.8	1.39	10.79	0.13	0.125	11.14	1.02	155.10	11.58	14.37	72.95	4.92	2.71	12.91
	W	Loam	44.4	35.9	19.6	6.7	9.05	63.32	0.60	1.205	10.55	0.50	447.21	28.96	7.06	167.13	26.68	1.73	97.20
	**Mean**		**32.1**	**49.9**	**18.1**	**7.2**	**4.8**	**34.3**	**0.3**	**0.51**	**11.2**	**4.5**	**442.4**	**18.8**	**17.4**	**103.1**	**12.5**	**4.0**	**47.4**
TOB (2,800)	N	Loam	60.3	32.8	6.8	7.4	0.91	5.39	0.10	0.019	7.67	5.42	51.45	10.34	9.55	45.38	4.96	13.67	7.59
	S	Loam	37.5	47.8	14.6	7.4	0.85	5.11	0.07	—	7.30	—	38.31	7.83	7.50	112.13	4.27	8.08	4.72
	E	Sandy loam	60.7	29.3	10.0	7.5	1.01	5.77	0.08	0.019	7.21	4.31	70.00	13.70	12.13	52.54	2.28	6.47	3.55
	W	Loam	46.8	43.7	9.2	7.7	0.74	4.34	0.12	—	6.42	—	170.87	15.29	39.37	73.64	3.50	5.87	7.81
	**Mean**		**51.3**	**38.4**	**10.2**	**7.5**	**0.9**	**5.2**	**0.1**	**0.01**	**7.2**	**4.9**	**82.7**	**11.8**	**17.1**	**70.9**	**3.8**	**8.5**	**5.9**
OLA (3,022)	N	Sandy loam	70.2	17.0	12.8	7.5	1.39	9.66	0.12	0.102	8.05	1.21	121.00	9.17	12.53	57.21	1.01	2.84	2.02
	S	Loam	46.7	27.9	25.4	7.6	0.43	2.54	0.06	0.036	12.90	1.78	15.67	1.04	6.17	38.33	1.17	4.21	1.48
	E	Loam	50.7	30.8	18.5	7.9	0.92	5.92	0.08	0.015	7.82	5.56	197.00	14.93	33.28	45.51	0.93	2.56	0.92
	W	Loam	35.6	39.7	24.6	7.7	0.36	2.19	0.06	—	13.06	—	48.95	3.69	22.35	41.06	0.93	4.72	1.73
	**Mean**		**50.8**	**28.9**	**20.3**	**7.7**	**0.8**	**5.1**	**0.1**	**0.038**	**10.5**	**2.9**	**95.7**	**7.2**	**18.6**	**45.5**	**1.0**	**3.6**	**1.5**

^†^Combustion method.

^††^Kjeldahl extraction.

^¶^MBC = Microbial biomass carbon.

^¶¶^MBN = Microbial biomass nitrogen.

SOC, MBC and MBN decreased with increasing altitude. Similarly, organic matter (OM) decreased with altitude, and the C:N ratio was lowest at TOB. The concentration of SOC ranged from 2.2 to 66.2 g C kg^−1^ at west aspects of OLA and ACU summits, respectively. MBC was highest at ACU west and CUS south, while MBN was highest in all aspects of ACU. Microbial biomass C decreased by approximately 50, 14 and 12% in CUS, TOB and OLA, respectively. The C_mic_:C_org_ ratio did not follow a clear pattern. In fact, the lowest and highest C_mic_:C_org_ values were obtained in the highest summit (Table 1). The significance of the lmm analysis for each soil variable is indicated in Table 2. The fixed effect summit yielded significant differences for most soil variables, except for Total P, C:N, inorganic C, percentage of sand and silt, and N:P and C:P ratios (Table 2).

**Table 2:** Results of the linear mixed model analysis for soil variables with summit as fixed effect and aspect as random effect.

Soil	Intercept	Summits			ANOVA	
variable		CUS	TOB	OLA		
pH	<0.00001	0.0142	0.007	0.0041	**0.0147**	*
OM	<0.00001	0.2199	0.002	0.0014	**0.0031**	**
SOC	<0.00001	0.2303	0.0025	0.0019	**0.0041**	**
Total N	0.0001	0.2716	0.0035	0.0027	**0.0059**	**
Total P	0.0207	0.5319	0.0882	0.1212	0.0767	NS
C:N	<0.00001	0.3108	0.7453	0.1172	0.3641	NS
MBC	<0.00001	0.2274	0.0016	0.0014	**0.0028**	**
MBN	<0.00001	0.0661	0.0101	0.0006	**0.004**	**
CaCO_3_	NS	0.1834	0.2134	0.0898	0.3225	NS
Sand%	<0.00001	0.2205	0.3662	0.4092	0.1556	NS
Clay%	<0.00001	0.2788	0.0009	0.6038	**0.0037**	**
Silt%	<0.00001	0.1824	0.8065	0.2385	0.1288	NS
Ca^++^	<0.00001	0.0088	0.0657	0.4937	**0.0369**	*
Na^+^	<0.00001	0.3863	0.0557	0.4583	**0.0427**	*
Mg^++^	0.0001	0.133	0.4052	0.0078	**0.0046**	**
K^+^	0.0001	0.9467	0.0798	0.0076	**0.0209**	*
N:P	0.0012	0.9643	0.1376	0.4479	0.3482	NS
C:P	0.0012	0.8661	0.1308	0.4934	0.3068	NS

### Potential soil N mineralization rates

Significant differences were observed for $N{H}_{4}^{+}$ concentration for the two factors considered (summits and aspect) and the interaction, while only the factor summit and the interaction was significant for $N{O}_{3}^{-}$ concentration (Kruskal-Wallis ANOVA, p < 0.001; Table 3). Concentrations of $N{H}_{4}^{+}$ were significantly higher in the two lowermost summits compared to the uppermost summits (Table 3), i.e. 11.9 and 9.5 mg kg^−1^ in the west aspects of ACU and CUS, respectively. The concentration of $N{H}_{4}^{+}$ in the west aspect of ACU was significantly different to the other aspects (p < 0.05, Tukey HSD test), while it was lower in the west aspects of the uppermost summits. $N{O}_{3}^{-}$ concentration was highest at CUS in the south aspect, i.e. 84.3 mg kg^−1^ (Table 3). In ACU, $N{O}_{3}^{-}$ concentration was significantly highest in the west aspect (p < 0.05, Tukey HSD test). In TOB summit, no significant differences were found for $N{O}_{3}^{-}$ concentration among aspects, and the lowest value was obtained at OLA, where it varied significantly among aspects (p < 0.05, Tukey HSD test).

**Table 3:** Soil mineral N ($N{H}_{4}^{+}$-N + $N{O}_{3}^{-}$-N) concentrations, net N mineralization and accumulative nitrification in samples collected in the topsoil of four summits at Ordesa National Park (mean ± standard error).

Summit	Aspect	${\boldsymbol{N}}{{\boldsymbol{H}}}_{{\bf{4}}}^{{\boldsymbol{+}}}$	${\boldsymbol{N}}{{\boldsymbol{O}}}_{{\bf{3}}}^{{\boldsymbol{-}}}$	Net N mineralization potential^†^	Net N nitrification potential ^‡^	Cumulative N nitrification^¶^
		<-------------- mg kg^−1^ -------------->				
ACU	N	3.90 (0.17)^b^	20.96 (3.63)^b^	7.48 (0.14)^b^	7.31 (0.20)^ab^	153.60 (4.16)^ab^
	S	3.59 (0.14)^b^	26.49 (2.57)^ab^	9.52 (1.31)^b^	6.88 (0.54)^b^	144.42 (11.32)^b^
	E	3.67 (0.16)^b^	23.30 (3.60)^b^	8.06 (0.41)^b^	7.8 (0.41)^ab^	164.96 (8.65)^ab^
	W	11.86 (2.28)^a^	67.01 (11.57)^a^	23.16 (0.78)^a^	8.57 (0.11)^a^	179.92 (2.37)^a^
CUS	N	2.97 (0.06)^b^	15.53 (0.80)^b^	8.74 (0.08)^b^	8.52 (0.04)^b^	178.87 (0.93)^b^
	S	3.66 (0.17)^a^	84.34 (8.95)^a^	30.21 (6.86)^a^	8.51 (0.10)^b^	178.62 (2.11)^b^
	E	2.72 (0.09)^b^	25.88 (2.52)^bc^	1.71 (0.10)^b^	0.63 (0.23)^c^	13.24 (4.86)^c^
	W	9.48 (3.06)^a^	60.62 (11.48)^ac^	9.37 (0.02)^b^	9.17 (0.05)^a^	192.65 (0.95)^a^
TOB	N	4.47 (0.64)^b^	26.96 (5.58)^a^	0.60 (0.08)^b^	0.55 (0.06)^bc^	11.55 (1.31)^bc^
	S	9.12 (1.77)^a^	24.22 (2.13)^a^	2.37 (0.44)^a^	1.87 (0.11)^a^	39.19 (2.31)^a^
	E	4.10 (0.26)^b^	43.01 (11.20)^a^	0.50 (0.09)^b^	0.26 (0.07)^c^	5.50 (1.57)^c^
	W	3.28 (0.09)^c^	20.05 (2.49)^a^	0.86 (0.06)^b^	0.73 (0.06) b	15.27 (1.34)^b^
OLA	N	3.63 (0.09)^a^	41.00 (4.29)a	0.53 (0.09)^ab^	0.30 (0.08) b	6.20 (1.71)^b^
	S	3.06 (0.08)^c^	7.08 (0.38)^bc^	0.33 (0.03)^a^	0.33 (0.03)^b^	6.86 (0.68)^b^
	E	3.31 (0.11)^ac^	9.16 (0.34)^ac^	0.84 (0.01)^b^	0.81 (0.01)^a^	17.12 (0.12)^a^
	W	2.62 (0.06)^c^	6.82 (0.29)^b^	0.83 (0.09)^b^	0.72 (0.09)^a^	15.19 (1.96)^a^

Values followed by different lowercase letters within the same column indicate significant differences after Kruskal-Wallis ANOVA post-hoc multiple comparisons (Tukey HSD test, p < 0.05) for individual summits.

^†^mg $N{H}_{4}^{+}$ + $N{O}_{3}^{-}$·kg dry soil^−1^·21 d^−1^.

^‡^mg $N{O}_{3}^{-}$ kg dry soil^−1^ 21 d^-1^.

^¶^Calculated for an incubation period of 21 days for all samples.

**Figure 1:**
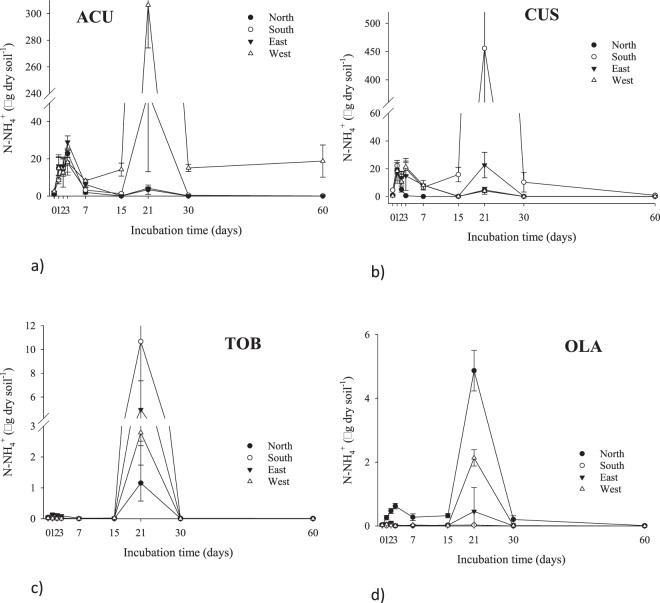
Temporal dynamics of soil $N{H}_{4}^{+}$-N (0–5 cm) in all summits: (**a**) Acuta, (**b**) Custodia, (**c**) Tobacor and (**d**) Olas. Data represent mean ± 1 S.D. The marks referred to incubation days from 1 to 60.

**Figure 2:**
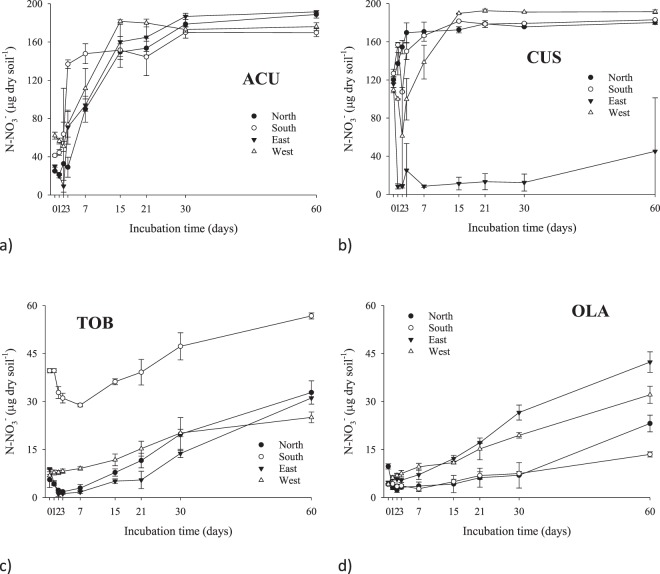
Temporal dynamics of soil $N{O}_{3}^{-}$-N (0–5 cm) in all summits: (**a**) Acuta, (**b**) Custodia, (**c**) Tobacor and (**d**) Olas. Data represent mean ± 1 S.D. The marks referred to incubation days from 1 to 60.

Regarding short-term $N{H}_{4}^{+}$ dynamics, low concentrations were observed and a flush of $N{H}_{4}^{+}$ was detected at day 21, decreasing till day 60 (Fig. 1); this pattern was repeatedly observed for the rest of soil samples collected in the different aspects. A possible reason for the decreasing trend of $N{H}_{4}^{+}$-N content during the period of day 5 to 14 may be due to bacterial N immobilization or nitrification. On the contrary, $N{O}_{3}^{-}\,$increased rapidly up to 15 days in soils from ACU and CUS, the two lower summits, reaching a plateau that lasted from 30 to 60 days (Fig. 2). The patterns were slightly different for soils collected in the two uppermost summits, the $N{O}_{3}^{-}$-N decreased to day 7 and slightly increased from day 7 onwards at TOB, while $N{O}_{3}^{-}$-N concentration decreased until day 3 and then increased until day 60 (Fig. 2).

The potential net N mineralization, i.e. the net change in $N{H}_{4}^{+}$ + $N{O}_{3}$ between fresh and incubated samples, was calculated only for 21 days to avoid artifacts from the experimental setup. Soils collected in the uppermost summits had lower N cycling rates than those from the two lower summits as revealed by the potential net N mineralization and nitrification which decreased significantly with altitude (Table 3). Net N mineralization was significant for all summits, i.e. ACU (F_3,11_ = 88.56, p < 0.001), CUS (F_3,11_ = 12.86, p = 0.002), TOB (F_3,11_ = 14.70, p = 0.001) and OLA (F_3,11_ = 12.56, p = 0.002). The net potential nitrification was also significant in the four summits, i.e. ACU (F_3,11_ = 4.15, p = 0.048), CUS (F_3,11_ = 977.38, p < 0.001), TOB (F_3,11_ = 77.13, p < 0.001) and OLA (F_3,11_ = 17.39, p < 0.001). The cumulative inorganic N in the topsoil increased in the lowermost summits with higher cattle presence. Significant differences were found between aspects at each summit (Kruskal-Wallis ANOVA, p < 0.05).

### Soil microbial community composition

Total PLFA biomass (total extracted lipid abundance) decreased with increasing elevation. Across summits it was noticeably higher in the lowermost summit (ACU) than in the rest of summits (Table 4), and ranged from 201.2 ± 110.7 nmol g^−1^ in the lowermost summit to 31.81 ± 17.5 nmol g^−1^ in the highest summit (average of all aspects). With regards to aspect, the highest PLFA biomass was observed in the western aspects in ACU, CUS and TOB, while in the uppermost summit (OLA), the highest PLFA biomass was found in the northern and eastern aspects (Table 4). In ACU west the functional groups Eukaryote, Gram negative, Gram positive, Actinobacteria, Fungi and arbuscular mycorrhizal fungi (AMF) had the highest abundance. Anaerobes were most abundant in ACU south. For all functional groups a general pattern was observed, a decrease in their abundance with increasing elevation (Table 4), with the highest values in the two lowermost summits, ACU and CUS.

**Table 4:** Functional groups of soil micro-organisms in soil samples taken (0–5 cm) in the four aspects of the studied summits at “Ordesa and Monte Perdido” National Park.

Summit	Aspect	Total PLFA	Functional group biomass^a^									
		biomass^a^	Eukaryote	Gram−	Gram+	Actinobacteria	Total Gram+	Total Bacteria	Fungi	AM Fungi	Total Fungi	Anaerobes
ACU	North	87.32	0.98	30.26	23.45	13.88	37.33	67.59	1.33	2.81	4.14	1.07
	South	217.55	6.08	82.54	62.66	21.10	83.76	166.31	5.76	7.14	12.90	1.81
	East	152.90	3.44	53.65	45.19	19.52	64.70	118.36	2.87	5.74	8.61	1.66
	West	346.89	6.67	151.32	93.43	28.56	121.99	273.31	5.56	12.42	17.98	1.44
CUS	N	78.36	2.10	27.51	17.86	13.57	31.43	58.94	1.23	3.23	4.46	0.74
	S	150.13	2.40	61.66	39.12	17.24	56.36	118.01	2.37	4.33	6.70	1.20
	E	49.41	1.35	18.20	11.28	7.50	18.78	36.97	0.85	1.78	2.62	0.37
	W	130.58	2.56	52.56	31.82	16.72	48.54	101.09	1.44	5.07	6.52	1.30
TOB	N	31.08	0.58	13.23	6.85	4.60	11.45	24.68	0.26	0.71	0.97	0.47
	S	28.03	0.82	10.84	7.46	3.18	10.64	21.48	0.27	1.12	1.38	0.19
	E	43.35	0.80	17.52	10.28	6.19	16.47	33.99	0.69	1.27	1.96	0.59
	W	62.04	1.17	21.26	18.12	6.63	24.75	46.01	1.88	3.72	5.60	0.83
OLA	N	47.42	0.59	19.20	10.61	7.91	18.52	37.72	0.52	1.53	2.05	0.65
	S	11.25	0.55	5.33	1.40	1.81	3.21	8.55	—	—	—	0.22
	E	45.34	1.65	17.00	10.36	7.54	17.90	34.90	0.75	1.28	2.02	0.56
	W	23.25	0.84	9.06	4.88	4.36	9.23	18.29	0.58	0.42	1.00	0.19

^a^Sum of all fatty acids in the sample (nmol g dry soil^−1^).

**Figure 3:**
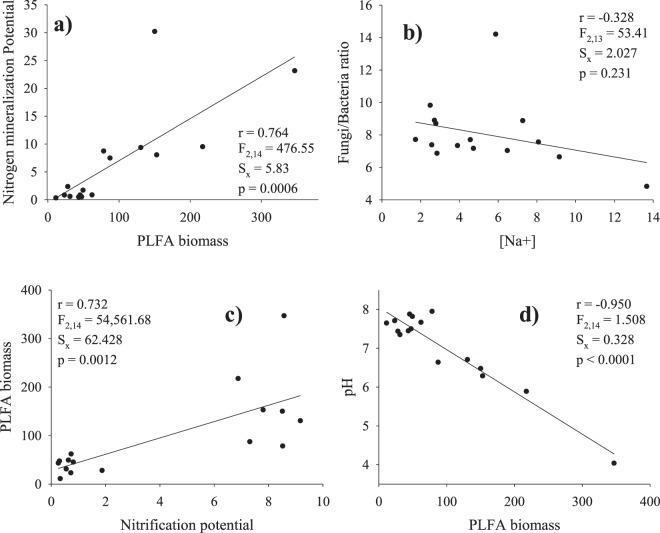
Fitted regression curves between PLFA biomass and Fungi/Bacteria ratio with selected soil variables: (**a**) correlation between PLFA biomass and nitrogen mineralization potential; (**b**) correlation for Na^+^ concentration in soil and Fungi/Bacteria ratio (not significant) (**c**) correlation between PLFA biomass and Nitrification potential, (**d**) idem for PLFA biomass and Nitrification potential and pH. Correlation coefficients (r), standard errors of estimates (sx) and F and P values are given. Initial soil variables and biomarkers were selected after multiple correlation analysis was performed (not shown). Fungal/biomass and Gram −/Gram+ ratios in soils collected in the four summits at “Ordesa and Monte Perdido” National Park.

When functional group ratios (fungal/bacteria and Gm+/Gm− bacteria) were correlated with soil variables no significant relationships appeared (Fig. 3). The fungal/bacteria ratio was lowest at TOB in the northern and highest in the western aspects, respectively. The Gm+/Gm− bacteria ratio was lowest in the southern aspect of OLA summit (0.26) than in the lowermost summit ACU, (0.62–0.84), reflecting the relative dominance of Gm−bacteria biomarkers at lower altitudes (not shown). Only PLFA biomass was positively correlated with nitrogen mineralization potential and nitrification and negatively correlated with soil pH (Fig. 3).

The most abundant PLFA biomarkers for the different functional groups (Supplementary Table S1) were representatives of Gram positive (Gm+) bacteria with the branched group with *iso* or *ante-iso* methyl branching such as *a*15:0, *i*15:0, *i*16:0, *a*17:0 and 18:1ω9c; Gram negative (Gm−) bacteria biomarkers such as 16:1 ω7c, 17:0 cyclo ω7c, 18:1ω7c, 18:1ω5c, and 19:0 cyclo ω7c; the biomarker 18:2ω6c (fungi) and 16:1ω5 for AMF. There were also representatives of actinobacteria like 10-Me 16:0, 10-Me 17:1ω7c, 10-Me 18:1ω7c, 10-Me 18:0 and unspecified saturated lipids 16:0 and 18:0. In general, the microbial community was dominated by biomarkers of Gm – bacteria and actinobacteria (at least 40% of the total fatty acids in all summits and aspects). The percentage of fungi in these soils was less than 5% of the total fungal PLFA biomass. The largest amount of fungal PLFA biomass was measured in TOB western aspect while no fungi markers were found in the southern aspect of OLA (Table 4). AMF contributed to a small percentage of the total fungal PLFA biomass. Except for soils collected at the western aspect of TOB, more abundance of fatty acid biomarkers for bacteria than for fungi were present (the fungal/biomass ratio was below one).

**Figure 4:**
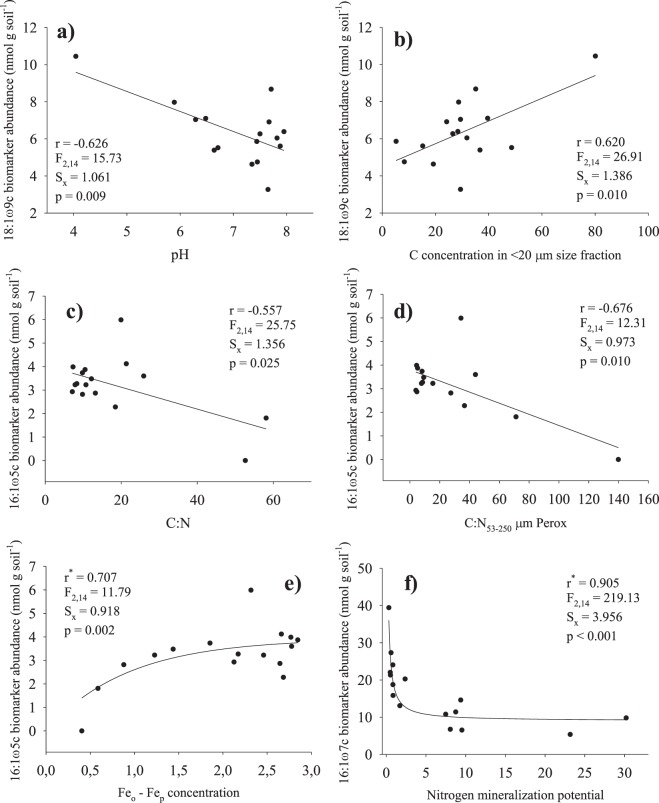
Fitted regression curves between fungal and AMF biomarkers 18:1ω9c, 16:1ω5c, 16:1ω7c and selected soil variables: (**a**) correlation between the abundance of 18:1ω9c biomarker and pH, (**b**) idem for 18:1ω9c biomarker and soil C concentration in the <20 µm size fraction, (**c**) idem for 16:1ω5c biomarker and C:N in the bulk soil, (**d**) idem for 16:1ω5c biomarker and the residues after peroxide attack of the 53–250 µm soil aggregates, (**e**) idem for 16:1ω5c biomarker and Fe_o_ – Fe_p_ mineral indicator (ferrihydrite) and (**f**) correlation between the abundance of 16:1ω7c biomarker and net nitrogen mineralization potential. Correlation coefficients (r), standard errors of estimates (sx) and F and P values are given. Initial soil variables and biomarkers were selected after multiple correlation analysis was performed (not shown).

A significant relationship was found for some PLFA biomarkers and different soil variables across the altitudinal gradient (Fig. 4). We also explored the relationship between the microbial community composition at sampled sites and soil C concentration in different particle size fractions (<20, 20–53 and 53–250 µm) and mineralogy. This could provide further insights into decomposition processes in these areas. The data were taken from Jiménez and Villar^29^. Actually, a clear relationship was highlighted between Fe_o_ – Fe_p_ indicator, i.e. the content of ferrihydrite mineral^30^. An inverse relationship was observed between fungi other than AMF, only for 18:1ω9c, and soil pH (r = −0.626, p = 0.009; Fig. 4a) and positively with C concentration in < 20 µm size fraction (r = 0.620, p = 0.010; Fig. 4b). Soil C:N ratio was negatively associated with the abundance of the AMF biomarker 16:1ω5c (r = −0.557, p = 0.025; Fig. 4c), and also for the C:N ratio of the residues of 53–250 µm particle size fraction after hydrogen peroxide attack (r = −0.676, p = 0.010; Fig. 4d).

Finally, the abundance of 16:1ω7c (biomarker for Gm− bacteria) was significantly correlated with N mineralization potential (Fig. 4f). The individual markers for Gm+ bacteria had a low predictive power when these were regressed together against N mineralization rates (data not shown).

### Multivariate statistics ordination

**Figure 5:**
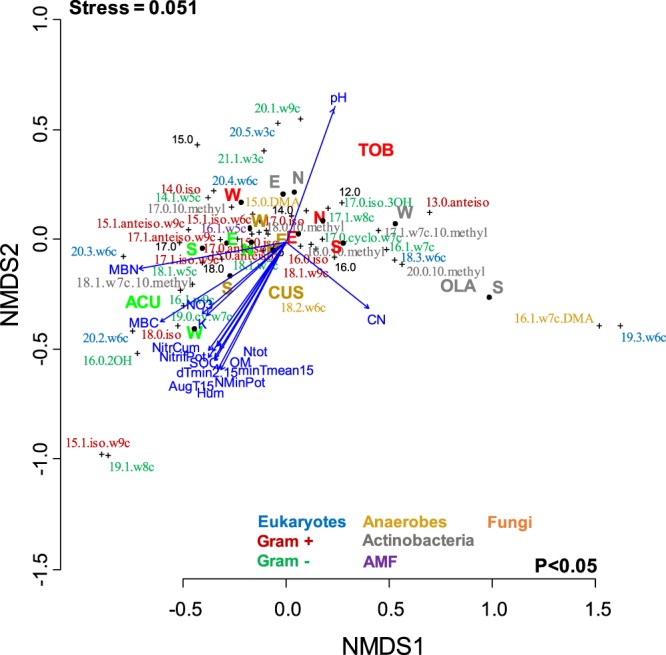
Non-metric multidimensional scaling (NMS) of soil microbial PLFA community ordination and its significant relationship with soil variables. Axes were arbitrary and scaled in Bray-Curtis dissimilarity units. The significance was based on 999 permutations and only significant variables (p < 0.05) are shown. Arrow length refers to the strength of correlation.

The NMS yielded a final stress value (i.e., departure from monotonicity between the distance measure and the distance in ordination space) of 0.051 for PLFA biomarkers (Fig. 5), which is considered an excellent representation in reduced dimensions^31^.

The NMS ordination (Fig. 5) showed that soil microbial composition differed with summits, the majority of the functional groups were present in all summits and aspects, although with different PLFA biomarkers. Gram negative bacteria (16:1ω9c and 16:0 2OH) were abundant in the lowermost summit, ACU, while anaerobes (16.1ω7cDMA) and Eukaryote (19:3ω6c) biomarkers were characteristic in the highest summit, OLA. The most abundant biomarkers at TOB, the second uppermost summit, were 20:5ω3c, 20:1ω9c and 21.1ω3c (Fig. 5).

Soil variables that were significantly (corrected p after FDR procedure) associated with the NMS ordination are listed in Table 5. Most of the significant soil variables were grouped around ACU west: SOC, OM, nitrification potential and N mineralization potential, MBC, MBN, Soil moisture, June T_av15_, June minT_av15_, June daily minimum T_02–15_, and AugT_15_, June daily minimum T_15_, June T_av2–15_, Total N, pH, $N{O}_{3}^{-}$ and K^+^. Soil pH and C:N were in opposition to the rest of soil variables. Actinobacteria were characteristic in the uppermost summit OLA together with specific PLFA biomarkers of anaerobes and eukaryotes.

**Table 5:** Correlation coefficients of soil and microbial community characteristics with the first and second axis of phospholipid fatty acid (PLFA) nonmetric multidimensional scaling (NMS).

Variable	r^2^	Initial p	P_corr_	
MBN	0.9060	0.001	0.002	**
MBC	0.8789	0.001	0.002	**
Soil moisture	0.7765	0.001	0.002	**
N mineralization potential (N Min_pot_)	0.7607	0.001	0.002	**
pH	0.7333	0.005	0.010	**
minT_av15_	0.7316	0.001	0.002	**
dTmin_2–15_	0.7175	0.001	0.002	**
minT_av2-15_	0.6912	0.001	0.002	**
AugT_15_	0.6878	0.001	0.002	**
OM	0.6702	0.001	0.002	**
dTmin_15_	0.6551	0.001	0.002	**
SOC	0.6536	0.001	0.002	**
T_av2-15_	0.6420	0.001	0.002	**
T_av15_	0.6351	0.002	0.004	**
Nitrification potential (Nitrif_pot_)	0.5922	0.001	0.002	**
N_tot_	0.5900	0.002	0.004	**
Cumulative N nitrification (N Cum)	0.5716	0.001	0.002	**
NO_3_^−^	0.4728	0.019	0.037	*
K^+^	0.4480	0.022	0.043	*
C:N	0.4386	0.027	0.052	NS
Mg^++^	0.3533	0.066	0.128	NS
CaCO_3_	0.3199	0.060	0.116	NS
Clay	0.2973	0.091	0.176	NS
P_tot_	0.2348	0.164	0.318	NS
NH_4_^+^	0.2004	0.229	0.444	NS
Sand	0.1727	0.305	0.591	NS
Silt	0.1086	0.464	0.899	NS
C:P	0.1041	0.458	0.887	NS
N:P	0.1041	0.478	0.926	NS
Ca^++^	0.0884	0.545	>1	NS
Na^+^	0.0151	0.905	>1	NS

The R^2^ denotes the proportion of explained variance and the p-value the significance of the correlations (p < 0.05). The corrected p* value indicates the probability at p = 0.05, after FDR procedure correction of p = 0.0031 (0.05/16) corrected associated one-tailed probability (p = 0.05) are indicated after the false discovery rate (FDR) procedure (Benjamini and Yekutieli 2001). PLFA relative abundance transformed data were used.

***0.001, **0.01, *0.05; probability correction with the False Discovery Rate (FDR) procedure was: P_corr_ = 0.05*31/16 (the 16^th^ ranked test yielded a p value lower than the initial p value; see text for explanation).

## Discussion

### Soil microbial community structure and hierarchical pattern

Studies on the variations of soil microbial community composition and activity along altitudinal/climatic gradients and associated vegetation zones are not abundant. The soil microbial community structure varies with increasing elevation in mountain soils^11,12,16^. Decreasing activity of microorganisms with increasing altitude in an Alpine environment was observed^32,33^, and the fungal population and relative amount of Gm – bacteria increased with increasing elevation^34^. Djukic *et al*.^4,25^ found that neither microbial community size nor activity showed a consistent elevation trend in a climosequence from the montane to the subalpine and alpine climate/vegetation in the Austrian Alps (from 900 to 1900 m a.s.l.) They showed that the fungal PLFA biomarkers were significantly higher in the forest sites (between 9.2 and 6.7 mol%) compared to the shrubland and grassland sites (between 4.5 and 2.3 mol%). They concluded that with the expected upward migration of the treeline with climate warming, the abundance of fungi in the higher zones will increase. In a study conducted in disturbed sites of US logged forests, the observed decrease in fungal abundance with increasing altitude was correlated with decreased nitrogen mineralization^35^. Although soil microorganisms may be well adapted to the prevailing climate conditions in Alpine areas, no clear patterns are observed. In a recent study by Nottingham *et al*. (2018) the diversity of soil microorganisms was mean annual temperature along an altitudinal gradient in the Peruvian Andes and to a lesser extent soil pH and moisture. The most important drivers explaining the microbial community composition along the gradient are linked to soil humidity and soil temperature, pH and OM (Table 5). Our analysis showed that the best soil T indicator was minT_av15_ and dTmin_2-15_.

In our study, the effect of aspect was demonstrated for soil microorganism composition, i.e., total PLFA biomass was higher in the western aspects of ACU and TOB while for CUS and OLA the highest PLFA biomass was obtained in the southern and northern aspects, respectively (Table 4). No general pattern was observed in which the lowest values of PLFA biomarkers are found in the north aspects of each summit; in the lowest summit, ACU, values were lowest in north aspect compared to the rest, while in CUS these were found in the east aspect; fungi were lowest in TOB north, while eukaryote biomarkers were lowest in the east aspect, gram negative and actinobacteria were lowest in the south aspect and gram positive and AMF in the north aspect; in OLA the lowest values were found in the south aspect. Other studies, like Huang *et al*.^36^ reported an effect of slope orientation on soil microbial community composition, with higher relative abundance of Gm – bacteria and aerobes on the shady slopes of the Chinese plateau (1,400 m a.s.l.) In a forest of the Western Carpathians, the soil microbial community structure indices calculated from PLFA data^1,37^ did not vary along an altitudinal gradient (600–1200 m a.s.l.), similar to data reported by Winkler *et al*.^21^. In our study, on the contrary, the reasons may be due to the rocky structure in the southern facing slope of this summit and the low abundance of plants, especially in the southern and western facing slopes (Supplementary Table S2).

The composition of the soil microbial community is known to be controlled by a set of environmental factors, like soil pH, C:N ratio and plant community composition gradient, although not for fungal biomarkers^25^. In our study we found that climatic factors (soil moisture and T) was the main factor in the hierarchy structuring the microbial composition across the summits, followed by pH and OM. This is in agreement with Lavelle *et al*.^38^ who reported that climatic and edaphic determinants are likely to be more important in temperate ecosystems. In our study the abundance of PLFAs in the bulk soil was determined by soil pH and C:N ratio and the relative abundance of AMF biomarker 18:1ω9c was negatively correlated with soil pH.

In general, our results demonstrate a fundamental role for environment, principally moisture, temperature and OM (Table 4) in explaining the pattern observed for the community composition of soil PLFA biomarkers. In recent study undertaken in the Peruvian Andes Nottingham *et al*.^39^ found that the dominant factor explaining α-diversity for soil biota groups along an altitudinal gradient was mean annual temperature. The difference with our study is that we found that the best T indicator to explain the pattern observed was minT_av15_, i.e. June Tmin of daily average T recorded during 2015, when field work was performed. We would have expected a stronger effect of soil T, but it seems that in these summits soil moisture is the most important driver affecting soil biota. Whether these differences are the result of variations among temperate and tropical sites, more studies are thus needed to further address this hypothesis.

Microbial composition and activity has been demonstrated to shift along gradients in alpine ecosystems^40^. In the latter study, the shift in microbial community composition, including functional groups of Gm−, Gm+, and bacterial to fungal ratio, was linked to soil C:N ratio and pH, which is also in agreement with Yao *et al*.^41^. Soil C:N ratio was also found to be positively correlated with soil bacteria and fungal biomass^37^. Some authors have reported a positive correlation between Gm− and fungi and organic N, while anaerobes and actinobacteria were positively correlated with total N^36^. SOC and total N seemed to be the most important driver of microbial biomass and composition, similarly to results reported by Yao *et al*.^41^, who found a close relationship between microbial biomass, fatty acids and OM in Chinese soils. An increase in $N{H}_{4}^{+}$-oxidizing bacteria with increasing fertility of grassland soils was reported over a period of 40 years^42^.

Soil C availability is known to affect microbial activity and biomass differently^43,44^. Microbial biomass C is closely related to the growth of vegetation and to changes in the microorganism content of the soil^36^. Total N and $N{O}_{3}^{-}$-N levels were shown to be positively related to MBC and the amount of anaerobes and actinobacteria. Cmic:Corg ratio relates to the microbial activity and its potential to mineralize organic substances^45^. An increase in the microbial activity could result in increasing the MBC and soil organic N levels that are required for plant growth through the C and N cycles^36^. The change in substrate quality across vegetation succession has been hypothesized to presumably promote faster growing bacteria (particular Gm− bacteria) versus fungal mediated decomposition cycles^46^. In our study, however, AMF may have a key role, especially in the soils at the highest summit.

### Soil chemical properties

The C:N and N:P ratios are considered important information source in ecosystems^47^. Nutrient limitation to plant growth, in particular, N and P availability has been shown to be major drivers of primary production. Plant productivity is limited in these alpine soils, i.e. the N:P or Redfield ratio, although difficult to interpret and rarely used in soil ecology, was below the threshold of 13.1 established by Cleveland and Liptzin^48^. Several studies with different plant communities have shown that N is limited when the N:P ratio is <14, and P is limited when the ratio is >16^49^. Plant productivity is reduced on northern aspects as temperature reduces, freezing periods and snow cover duration increase. This process determines C inputs to the soil, which can be low in alpine-nival environments^50^. In southern aspects, milder temperatures and shorter period of snow cover may allow slightly higher production of plant biomass.

A C:N ratio of <30 is considered as predictor for N mineralization while C:N ratios above this values can be used as predictor for initial net N immobilization^51^; in our study, soil N availability decreased in the uppermost summits. A decline of soil organic matter with increasing altitude was also reflected by decreasing concentrations of total C in soil that led to a narrowing of C:N ratios with increasing altitude, especially in TOB summit (Table 2).

### N dynamics, mineralization and nitrification potential

Most plant available N ($N{H}_{4}^{+}$-N and $N{O}_{3}^{-}$-N) in natural ecosystems is the result of OM decomposition^52^. N mineralization or ammonification ($N{H}_{4}^{+}$-N) is the immediate product of this process, while nitrification (the conversion of $N{H}_{4}^{+}$-N to $N{O}_{3}^{-}$-N) is performed by specific groups of microorganisms^53^. Nitrification, is one of the key microbiological processes in the soil N cycle and is regulated mainly by temperature, soil moisture, input rates and quality of plant residues, as well as SOC availability^54^. Nitrification processes are altered by a set of abiotic and biotic factors, which can thus impact on N_2_O emissions to the atmosphere, which is why estimating nitrification potential is essential.

Some studies have reported that microbial biomass prefer $N{H}_{4}^{+}$ to $N{O}_{3}^{-}\,$^55,56^, particularly when pH ranges between 6 and 7. The high variability observed in $N{H}_{4}^{+}$-N and $N{O}_{3}^{-}$-N concentrations and incubation data indicates a spatial and temporal dependence of soil nutrient dynamics in these summits, which are probably dependent of several factors (not specifically analysed in this study). Changes in ecosystem N dynamics along an altitudinal gradient in alpine-nival ecotone was demonstrated by Huber *et al*.^24^. They reported data from *Caricion curvulae* alpine pasture with nival plant groups of *Androsacion alpinae* on the southwest aspect of Mt. Schrankogel (Tyrol, Austria). The concentration of $N{H}_{4}^{+}$-N and $N{O}_{3}^{-}$-N ranged between 4 and 2 µg N g soil^−1^, respectively (from 2,906 to 3,079 m a.s.l., siliceous parent material). In our study, although the altitudinal variation is larger, almost 900 m between the lowest summit ACU and the highest summit OLA, similar values of $N{H}_{4}^{+}$-N concentration were obtained. The concentration was only higher in some aspects, between 9.1 and 11.9 µg N g^−1^ soil from the west facing slopes of ACU and CUS and the south facing slope of TOB (Table 2). In general, the two lower summits had higher concentrations of extractable N relative to the uppermost summits. In addition, low $N{H}_{4}^{+}$-N concentrations were found in the summits, resulting in limitations for mineral N uptake by plants, a limitation that can be linked to reduced microbial activity both in terms of biomass and number of PLFA biomarkers (Fig. 5). The lowest concentration of ions with increasing altitude shows that N mineralization rates decrease along the gradient, as observed by Huber *et al*.^24^. By contrast, the $N{O}_{3}^{-}$-N concentrations were much higher in our study, with a maximum of 84.3 µg N g^−1^ soil, than those obtained by the same authors, between 1 and 3 µg N g soil^−1^, and only in OLA summit differences were not markedly evident (Table 2). This indicates that nitrification is an important process in these summits, although this hypothesis should be further tested.

Under aerobic conditions $N{H}_{4}^{+}$-N can be rapidly used for microorganisms and be mineralized into $N{O}_{3}^{-}$-N for plant available N. The concentrations of $N{H}_{4}^{+}$-N in soils decreased with elevation reflecting decreasing rates of N mineralization with altitude. In our study, the highest $N{H}_{4}^{+}$-N concentration was 11.9 mg kg^−1^ in but in most summits and aspects the concentration was less than 5 mg kg^−1^, which might be related to the accessibility of $N{H}_{4}^{+}$-N by soil microorganisms. In the lowest summit no significant differences in the soil $N{H}_{4}^{+}$-N concentration were found between the northern, southern and eastern aspects (Table 4). As hypothesized, at the highest summits (TOB; OLA) the rates of net N mineralization and nitrification potential were lower than in the lowermost summits (ACU; CUS). Less available soil N was present at the higher summits.

C and N cycling processes like nitrification and N mineralization rates responded significantly to warming in mountain areas^57,58^. The expected increased temperatures can therefore profoundly influence soil processes in these areas by increasing nutrient availability^59^ and enhancing rates of N mineralization^60,61^, although climate manipulation studies have shown that microbial community structure was not affected at mid-term (4 years) in subalpine grassland soils^62^.

## Conclusion

The data presented support the conclusion that concentration of elements decreased with increasing altitude, and that highest nutrient concentrations were obtained in the southern aspects of summits irrespective of altitude. Soil microbial biomass was highest in the lowermost summit. A set of hierarchically organized factors which regulate microbial composition and activity with climate (soil T) as the main factor structuring the microbial composition across the summits followed by OM and plant cover. Clear relationships were found between microbial community biomarkers and selected soil variables like pH, C:N soil mineralogy and N mineralization potential. The use of PLFA analysis in combination with NMS multi-variate statistics and linear mixed models has resulted in providing a clear fingerprint of the microbial community structure across summits. Although it is difficult to identify the individual influence of those biotic and abiotic parameters on the microbial community, the different summits and aspects were clearly separated in the multi-variate ordination.

As hypothesized in other studies, if the projected upward migration of plants in alpine environments due to thermophilization process is taking place, this will result in changes in the microbial community composition and therefore patterns of decomposition rates are expected to vary. The low abundances of functional groups (AMF, Eukaryote, Anaerobes, Fungi) in the uppermost summits may well be considered as indicators to follow changes linked to climate warming. Further studies are needed to explore the influence of above- and belowground biotic and abiotic factors on soil processes driven by microbial communities in these areas.

## Methods

### Site description

The study was conducted in Monte Perdido Massif, which is part of the Long-term Ecological Research (LTER) network, in four summits included in a pilot study area of GLORIA (Global Observation Research Initiative in Alpine Environments): “Punta Acuta” (ACU), 2,242 m; “Custodia” (CUS), 2,519 m; “Tobacor” (TOB), 2,779 m; and “Punta de las Olas” (OLA), 3,022 m a.s.l. (see Supplementary Fig. S1). All summits are within the area of the National Park, which is a Biosphere Reserve and World Heritage Site (UNESCO), and represented a gradient from the subalpine (2,242 m a.s.l.) to nival (3,012 m) vegetation belts and zonation with respect to altitude. Yearly average precipitation and temperature in the area are 1,720 mm and 5 °C, respectively (last 29 years at 2,200 m a.s.l.)^63^. The soil parent material comprised mainly calcareous substrates such as sandstone at ACU, CUS (the lowermost summits) and TOB and limestone at OLA (the uppermost ones). Soils are defined as humic Dystrocryept (Inceptisol) in the lowermost summits, and lithic Cryorthent (Entisol) in the uppermost summits.

Extensive domestic grazing (goat, sheep and cattle), primarily during the summer, has taken place for centuries, and is currently a permitted activity within the park. As a result, the upper tree line is ca. 300 m below its potential altitude as determined by climate^64^. Only scattered groups of Mountain pine (*Pinus uncinata* Ramond ex DC.) approach summit ACU at 2,180-2,200 m and no trees or shrubs are observed above.

The most dominant species in each summit are listed below:

ACU (lower alpine belt) – Festuca gautieri (Hack.), Festuca eskia Ramond ex DC.; Achillea millefolium L., Geranium cinereum Cav., Arenaria purpurascens Ramond ex DC. and Trifolium alpinum L.;

CUS (lower alpine belt) – Helictotrichon sedenense (Clarion ex DC.) Holub; F. gautieri (Hack.); G. cinereum Cav.; Poa alpina L; A. purpurascens Ramond ex DC.; Galium pyrenaicum Gouan, Lotus corniculatus L.; Trifolium thalii Vill. in CUS;

TOB (upper alpine belt) – Leucanthemopsis alpina (L.) Heywood; A. purpurascens Ramond ex DC.; G. pyrenaicum Gouan; Saxifraga oppositifolia L., H. sedenense (Clarion ex DC.) Holub; Thymus praecox Opiz; Saxifraga moschata Wulfen; Poa alpina L; G. cinereum Cav.; Festuca pyrenaica Reut.; T. thalii Vill.; Crepis pygmaea L.; Linaria alpina (L.) Mill.;

OLA (subnival belt) – Androsace ciliata DC.; S. oppositifolia L.; Saxifraga pubescens Pourr. subsp. iratiana (F.W. Schulz) Engl. & Irmscher; P. alpina L; Silene acaulis (L.) Jacq.; C. pygmaea L.; L. alpina (L.) Mill., Pritzelago alpina (L.) Kuntze; Minuartia cerastiifolia (Ramond ex DC.) Graebn.

While hiking activities and alpinism are normally present, there is little evidence of herbivory at the upper elevations.

### Soil sampling

The sampling protocol is depicted in supplementary Fig. S2. All soil samples were taken in each aspect of the four summits (ACU, CUS, TOB, OLA) 15 m below the summit point, as vegetation inventories are conducted every 7 years within the GLORIA network^65^. Precautions were thus adopted to reduce human disturbance pressure in this nature reserve like avoiding excessive trampling in the area and reducing impact when soil cores were taken. Temperature loggers were installed in the topsoil (0–10 cm) of all summits and aspects in the summer of 2001.

Different soil samples were taken in this study based on the required explicative variables:

- For classical soil physico-chemical analyses, a 500 g bulk soil sample was taken. Soil was first air dried and later sieved at 2-mm after visible root fragments and stones were manually removed.
- Four soil samples (0–5 cm) were taken during the months of July in ACU,CUS and TOB summits and August in OLA (due to the presence of snow) of 2015 in each of the four cardinal points of each summit, i.e. North, South, East and West, following a transect of at least 10 m in each aspect (supplementary Fig. S2). This group of soil samples were transported to the lab in an ice cooler and stored at 4 °C to stop soil mineralization processes. These samples were used for microbial biomass carbon (MBC) and nitrogen (MBN) determinations and mineral N ($N{H}_{4}^{+}\,$ and $N{O}_{3}^{-}$) dynamics.
- Another set of four soil samples (0–5 cm) were taken within a radius of 10 m from which a composite sample was obtained and stored in plastic bags. These were preserved in a cooler in the field and transported the same day to the laboratory where they were sieved at < 2 mm mesh and then lyophilized (freeze-dried) and stored at −20 °C. These samples were used for microbial community characterization with phospholipid fatty acid (PLFA) analysis.

At the time of sampling an inventory of the present plant community was performed in each aspect of each summit. The inventory was performed 10 m below the established highest summit point to obtain a vegetation abundance/dominance index (Supplementary Material Table S2). More information about the vegetation sampling can be found in Pauli *et al*.^65^.

### Soil physical and chemical properties

Soil pH in water (1:1 mass:volume ratio) was determined in air-dried < 2 mm sieved soil with a glass electrode. Soil texture was determined with the pipette method^66^ and total N with the Kjeldahl method^67^. Exchangeable base cations (Na^+^, K^+^, Ca^2+^ and Mg^2+^) and P (Mechlich) concentrations were analyzed by ICP-OES (Inductively Coupled Plasma Optical Emission Spectrometry, iCAP 6300 DUO, Thermo Electron Corporation, UK) after acid-digestion with hot HNO_3_. The gravimetric soil water content was calculated on a dry soil basis^68^ as:

$$[(\mathrm{Wet}\,{\rm{soil}}\,{\rm{weight}}-{\rm{dry}}\,{\rm{soil}}\,\mathrm{weight})/\mathrm{dry}\,{\rm{soil}}\,{\rm{weight}}]/100$$

The dry combustion method was used for assessment of C and N concentrations (0.5-g finely ground <200 μm subsamples) in a VarioMax CN Analyzer (Elementar GmbH, Hanau, Germany). Inorganic C was measured by CO_2_ evolution with HCl^69^, and soil organic carbon (SOC) was determined by subtracting the total inorganic C from total C. When inorganic C was not present total C was considered as total SOC.

The C:N and N:P ratios were also determined, the former depends on the plant community present^56^, while the second, the Redfield ratio, relates to plant productivity in a particular ecosystem^70^.

### Microbial biomass C and N

The chloroform–fumigation extraction (CFE) method^52^ was used for determination of microbial biomass C and N. Total organic carbon (TOC) in fumigated and non-fumigated root-free soil subsamples was measured in 50 ml 0.5 M K_2_SO_4_ extracts of 25 g fresh soil (Shimadzu 500 TOC Analyzer). TOC in the non-fumigated extracts was assumed to be equal to the labile, extractable C pool. The difference in the flush of dissolved organic C between non-fumigated and fumigated samples (soil solution) allowed to estimate MBC after a proportionality constant (*Kc* = 0.38) was used. MBN was determined as the difference between extractable N in fumigated and non-fumigated samples using a correction factor (*Kn*) of 0.54^71^. Values were converted to a per gram soil dry weight (dw) basis^52^.

The C_mic_:C_org_ ratio relates to the microbial activity and its potential to mineralize organic substances^45^.

### Soil microbial community structure

Phospholipid fatty acids (PLFA) are used to characterize microbial community structure^72–76^. PLFAs were extracted from 2 g of lyophilized soil, separated and methylated^77^. The resulting fatty acids methyl esters (FAMEs) were separated by gas chromatography using an Agilent 7890 A GC System equipped with a HP-ULTRA 2 column (length 25 m, ID 0.20 mm; J&W Scientific Inc.) and a flame ionization detector. The individual FAME peaks were identified and quantified with the software Sherlock™ PLFA Method and Analysis Package (MIDI, Inc., Newark, DE) The internal standard 19:0 phosphatidylcholine (Avanti Polar Lipids, Alabaster, AL, USA) was used for quantification of FAMEs. Although most of bacterial PLFAs have acyl chain lengths of between 14 and 20 carbons, there are however fatty acids longer than 20 carbons that are considered to be predominantly of bacterial origin or from micro-eukaryotes^78,79^, like 21:0, 22:0, 22:5 ω3, 22:6 ω3, and 24:0). This software was designed to detect such fatty acids in soil samples, and therefore they were also taken into account in our study.

The viable microbial biomass was calculated by summing PLFAs concentrations and reported as nanomoles of PLFA per gram of soil. Specific PLFAs were used as biomarkers to quantify the relative abundances (mol%) of particular microbial groups^80^.

Several PLFAs may have various sources^81^. Fatty acids indicating fungi and arbuscular mycorrhizae fungi (AMF) were summed as total fungal biomass. The bacterial biomass was calculated from the residual fatty acids which could be assigned to bacterial groups. Biomass was expressed relative to dry weight of the freeze-dried soil. The ratios of fungal/bacterial (18:2ω6 for fungi) and Gram positive/Gram negative bacterial markers were also obtained. Detailed information on the PLFA pattern of soil microorganisms can be consulted^76,82^. We did not calculate diversity indices since there are flawed approaches and are not recommended^81^ although recent studies continue to use it^83^. There are essential reasons why diversity calculations should not be based on PLFA data. This is especially important when dealing with Fungi which have very few different PLFAs in their membranes. There can be thousands of different fungal species in an only be able to detect ten major types of fungal PLFAs^81^.

### Mineral N and soil incubation for $N{H}_{4}^{+}\,$and $N{O}_{3}^{-}\,$determinations

Total N was determined with the Kjeldahl method. Soil mineral N content (plant available N, N_avail_) was extracted by shaking 4 g of subsamples of fresh soil stored at 4 °C with 40 ml of 1 m KCl solution for 30 min and after a 3-week incubation at room temperature while keeping soil moisture constant. The suspensions were later filtered and the extracts stored at −15 °C before analysis. The concentrations of ammonium ($N{H}_{4}^{+}$) and nitrate ($N{O}_{3}^{-}$) were determined in triplicate with standard colorimetric methods^84^. Water-soluble organic N was calculated as the difference between water-soluble N and inorganic N ($N{H}_{4}^{+}$ plus $N{O}_{3}^{-}$) in deionized water extracts.

Potential net mineralization was calculated as the net change in $N{H}_{4}^{+}$ and $N{O}_{3}^{-}$ between fresh and incubated samples, while potential net nitrification was calculated as the net change in $N{O}_{3}^{-}$, and time (t) the number of days in lab incubation. Accumulative net N mineralization and nitrification were calculated by summing the day-to-day accumulation of mineral N and concentrations in the incubated soils, respectively, and using the incubation days between the current sampling date and the previous sampling date, i.e., [$N{H}_{4}^{+}$ + $N{O}_{3}^{-}$]_0_ and [$N{H}_{4}^{+}$ + $N{O}_{3}^{-}$]_21_ are the respective concentrations of $N{H}_{4}^{+}$ + $N{O}_{3}^{-}$ (mg N kg soil^−1^) at t 0 and t 21 days. Soil fresh samples served as the initial samples for the course of incubation^85^.

### Statistical analysis

In our study classical statistical analyses like ANOVAs were not possible for some variables. No independent samples were available since each soil sample was collected from a single monitoring unit, i.e. every aspect in each of the four summits and, thus, no degrees of freedom were left over for inference. Only for $N{H}_{4}^{+}$ and $N{O}_{3}^{-}$ determinations 4 field repetitions were available for each aspect of each summit. In this case, a two-way non-parametric Kruskal-Wallis ANOVA was performed with summit and aspect as main fixed factors between groups for soil $N{H}_{4}^{+}$ and $N{O}_{3}^{-}$ concentrations and specific incubation dates. When significant differences appeared the Tukey’s honestly significant difference (HSD) test at α = 0.05 was used for mean comparisons.

In mountain summits, there are rocks, cracks, fissures, etc. and this constraints the number of available areas for soil sampling. This pseudorreplication issue^86^ is not due to the study design but to the site conditions. In such case, for those samples that do not constitute real replicates, like in the case of PLFA determinations, a composite sample was used to capture the local variability. Linear mixed model analyses were used^87^ with summit as fixed effect and aspect as random effect (one datum for each aspect, 4 data for summit) for soil variables of which no true replicates were available. Aspect is nested within summit, i.e., within each summit there are 4 aspects (N, S, E, W).

### Use of soil temperature (T) data

A correlation exists between a thermic vegetation indicator and habitat temperature across a range of European mountains^19^. They specifically showed that air temperature (T) in June was the best indicator. The strength of the correlation in our study area (ES-CPY) was in the limit of the significance (Spearman’s correlation rho = 0.6, p = 0.047). since dataloggers were installed on 31^st^ July 2001 and recorded every hour we were able to use in the multivariate analysis 7 variables related to soil T in June (0–10 cm):

1. Average T of June for the period 2002–2015 (T_av2–15_);
2. Average T of June in 2015 (T_av15_);
3. June Tmin of daily average T in 2015 (minT_av15_);
4. June Tmin of daily average T for the period 2002–2015 (minT_av2-15_);
5. Average of daily Tmin in 2015 (dTmin_15_);
6. Average of daily Tmin for the period 2002–2015 (dTmin_2-15_);
7. August mean soil T in 2015 (AugT_15_);

Data were averaged over June mean T values for the period 2002–2015 (available data), as this variable showed the best correlation. It is the temperature in the first part of the growing period which is most decisive for plant growth^88^. In the analysis we also used monthly mean T as two uppermost summits were not free of snow in June. Since we had all the data collected from the temperature loggers in all aspects and summits we wanted to precisely identify which variable of June temperatures could be the best indicator that influence microbial community composition and activity.

### Multivariate ordination

Non-metric multidimensional scaling (NMS) is an iterative multivariate ordination technique that is suited to non-linear data that are not normally distributed^31^. The ordination is based on rank order information in a dissimilarity matrix to identify a space of minimum dimensionality where a satisfactory large number of the inter-point distances (corresponding to the dissimilarities) between every object is best represented geometrically^89,90^. These distances are regressed against the original distance matrix and the predicted ordination distances for each pair of samples is calculated. This regression fit (after 999 permutations) is called stress (scaled from 0 to 1) and reflects how well the ordination summarizes how far the distances in the reduced-space configuration are from the original distances^91^. A good rule of thumb is when stress values is <0.2, i.e., it provides an excellent representation in the reduced dimensions of the analysis.

NMS was performed on relative lipid abundance data (mol%) in order to eliminate the total PLFA biomass effect and focus our analysis on relative changes in microbial community structure. Only PLFAs with concentration higher than 0.5 mol% were used in the analysis^74^. PLFA relative abundances were used in NMS after arcsine transformations to meet normality assumption.

### Probability correction

In both NMS analyses soil environmental and temperature variables (see below) were fitted to the PLFA ordination to search for correlations (after 999 permutations). Each test in the NMS yielded an initial *p* value which is then used to perform the corrections and search for significant differences at the corrected probability level. We used the false discovery rate (FDR) procedure^92^ to address multiple testing problems, i.e., as a mechanism to control the proportion of significant results that are in fact type I errors (‘false discoveries’) while simultaneously allowing type II errors to be reduced^93^. The procedure to adjust the significant α < 0.05 probability level is as follows:

$${P}_{i}\le (\frac{\alpha }{m})\ast i$$

where m is the number of tests and i is the test ranked in ascending order, i.e. *P*_(1)_ ≤…..≤*P*_(m)_. Starting with the highest p value each p is checked for this requirement; at the first p that meets the requirement its corresponding null hypothesis and all those having smaller p’s are rejected.

In our case, the final α’ corrected probability level was 0.026: *P*_*corr*_ = 0.05*31/16 (the sixteenth ranked test yielded a *p* value lower than the initial *p* value).

All analyses were conducted with different packages included in the R statistical software^94^. The metaMDS function in the package *vegan*^95^ in R were used and the package mice was used to deal with NAs in the data matrix of soil temperatures. Graphical displays were performed with Sigmaplot software version 13.0 (Systat Software, Inc.).

## Supplementary information

Supplementary Figures

Supplementary Info_Tables
