# Supplementary material for: Hierarchical drivers of soil microbial community structure variability in “Monte Perdido” Massif (Central Pyrenees)
Source: Sci Rep. 2019 Jun 19;9:8768. doi: 10.1038/s41598-019-45372-z (PMC6584728; doi:10.1038/s41598-019-45372-z)
Supplement: Supplementary file 1 — Supplementary Figures [file 41598_2019_45372_MOESM1_ESM.pdf]

Hierarchical drivers of soil microbial community structure variability in “Monte Perdido” Massif (Central Pyrenees)

Juan J. Jiménez, José M. Igual, Luis Villar, José L. Benito-Alonso & Jesús Abadias-Ullod

Supplementary Figure 1. Digital Elevation Map of the study area with the four summits included in the LTER "Monte Perdido" massif. The map was originally created in ArcGIS v. 10 (www.esri.com) by M. Gartzia (IPE-CSIC).

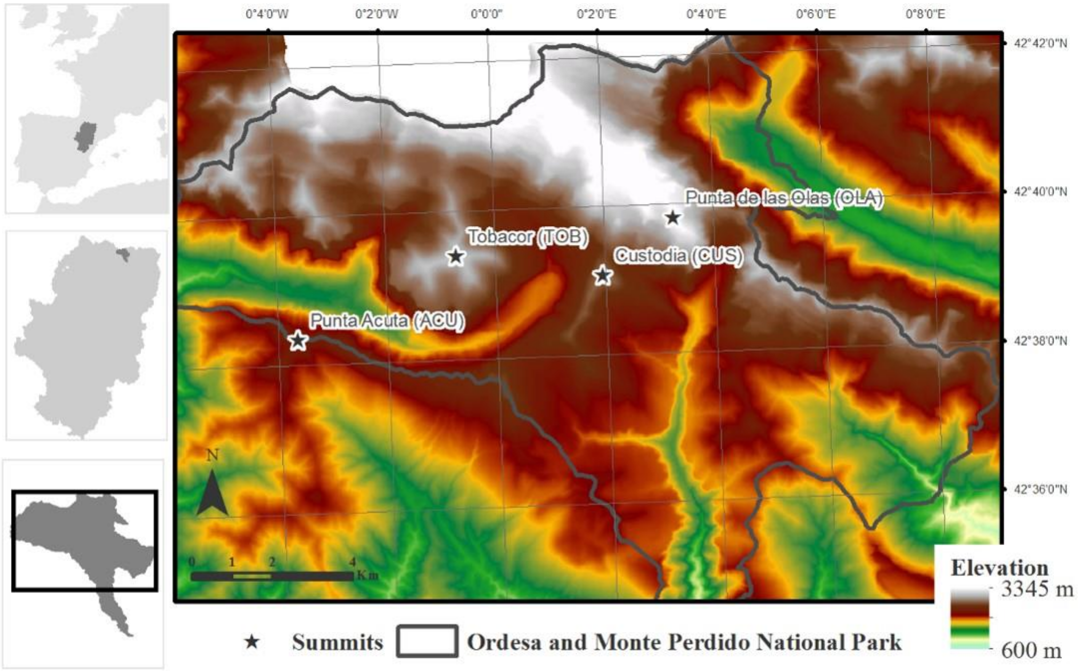

Supplementary Figure 2. Experimental design and soil sampling strategy.

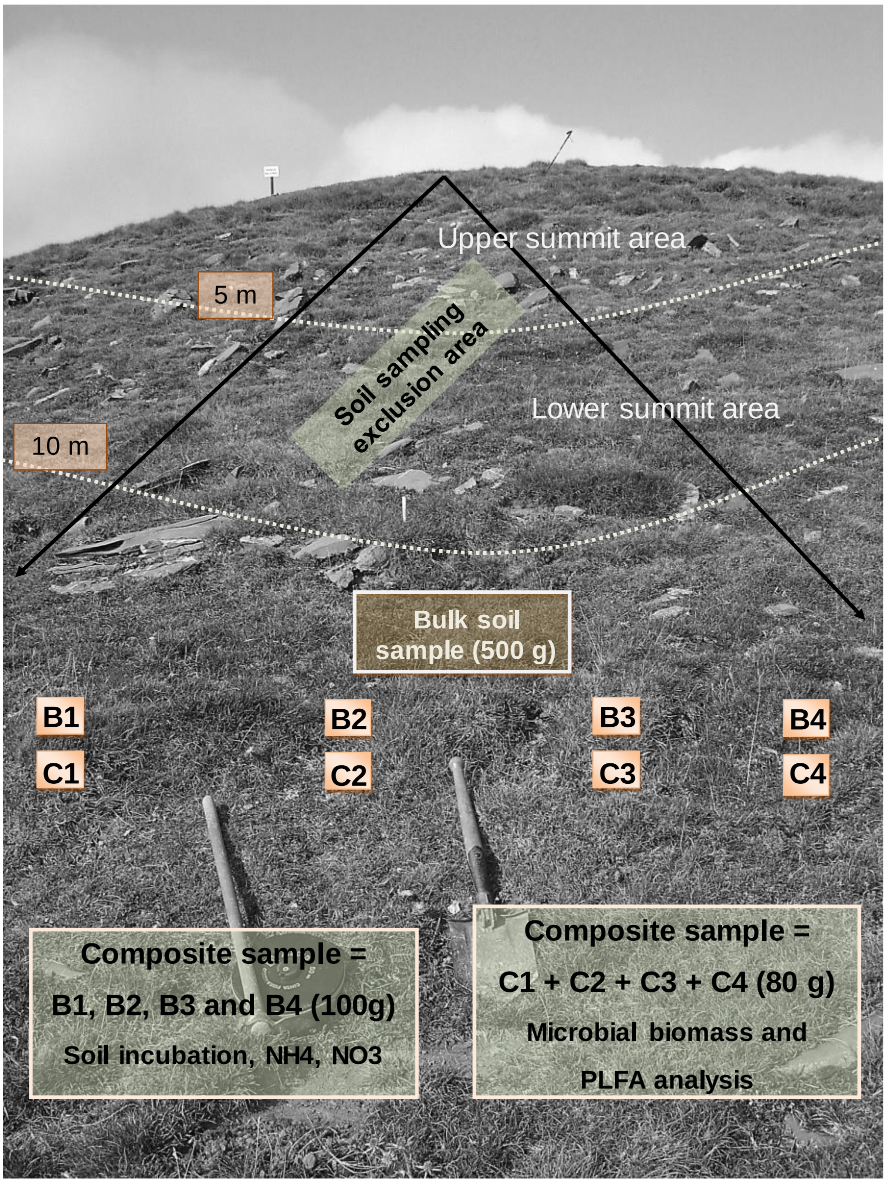

Picture from J.J. Jiménez
