# Supplementary material for: Hierarchical drivers of soil microbial community structure variability in “Monte Perdido” Massif (Central Pyrenees)
Source: Sci Rep. 2019 Jun 19;9:8768. doi: 10.1038/s41598-019-45372-z (PMC6584728; doi:10.1038/s41598-019-45372-z)
Supplement: Supplementary file 2 — Supplementary Info_Tables [file 41598_2019_45372_MOESM2_ESM.pdf]

Hierarchical drivers of soil microbial community structure variability in “Monte Perdido” Massif (Central Pyrenees)

Juan J. Jiménez, José M. Igual, Luis Villar, José L. Benito-Alonso & Jesús Abadias-Ullod

Supplementary Information

Table S1. Relative abundance of bacterial, actinobacterial and fungal phospholipid fatty acids (PLFAs) in soils (0-10 cm depth) collected in the four aspects of the studied summits at "Ordesa and Monte Perdido" National Park (Central Pyrenees).

PLFA	Summit															
	Acuta (2,232 m)				Custodia (2,519 m)				Tobacor (2,769 m)				Olas (3,012 m)			
	North	South	East	West	North	South	East	West	North	South	East	West	North	South	East	West
12:0	1.006	0.767	0.895	0.491	0	0	0	0.726	0.347	0.629	0.275	0.325	0.461	0	0.376	0.424
*a*13:0	0.359	0.398	0.287	0.389	0	0	0	0.345	0.215	0.285	0.193	0.239	0.205	0	0.208	0.321
*i*14:0	0.681	2.044	1.252	0.782	0.221	0.613	0	0.999	0	0	0.209	0.516	0.266	0	0.259	0
14:1 w5c	0	0	0.279	0.402	0	0	0	0.284	0	0	0	0	0.198	0	0.000	0
14:0	0.774	2.253	1.210	2.097	0.493	0.892	0.364	1.246	0.490	0.395	0.496	0.812	0.630	0	0.582	0.400
*i*15:1 w6c	1.642	2.850	2.591	0	1.047	1.487	0.473	2.327	0.522	0.565	0.891	1.229	0	0	0.749	0
*a*15:1 w9c	0	0.460	0	0	0	0.796	0	0.424	0	0	0	0.381	0	0	0	0
i 15:0	4.927	10.322	8.497	16.595	3.637	5.835	2.443	6.941	1.367	1.432	2.042	3.678	2.194	0.340	2.173	0.731
*a*15:0	5.309	14.626	10.583	16.072	2.508	8.576	1.853	5.935	1.195	1.886	1.791	3.454	1.860	0.287	1.675	0.542
15:0	0.356	1.042	0.623	0.924	0.277	0.672	0	0.526	0	0	0	0.448	0	0	0	0
15:0 DMA	0.931	1.806	1.541	1.437	0.738	1.202	0.375	1.195	0.356	0	0.437	0.666	0.557	0	0.448	0
*i*16:0	1.829	5.392	3.547	6.459	1.640	3.541	0.980	2.477	0.732	0.649	1.083	1.369	1.058	0.251	0.949	0.574
16:1 w9c	1.339	2.983	2.211	5.256	1.108	1.738	0.589	1.910	0	0	0	0	0	0	0	0
16:1 w7c	9.431	14.316	10.401	19.228	8.946	14.823	6.467	19.162	8.495	5.684	9.559	9.836	10.117	4.435	8.506	5.594
16:1 w5c	2.813	7.139	5.736	12.423	3.228	4.327	1.778	5.073	0.708	1.117	1.270	3.715	1.529	0	1.276	0.420
16:0	8.401	21.146	13.191	33.280	7.902	14.472	5.886	12.996	3.540	2.888	4.851	5.965	4.882	1.927	4.658	2.106
16:1 w7c DMA	0.141	0	0.115	0	0	0	0	0.109	0.113	0.187	0.152	0.162	0.094	0.224	0.110	0.194
10-Me 16:0	8.481	10.395	10.831	12.079	6.810	7.261	4.774	10.253	2.790	2.124	3.623	4.013	4.161	0.947	4.131	1.633

*i*17:1 w9c	0.559	1.118	1.145	4.927	0.625	0.903	0.411	0.823		0.400	0	0.280	0.497	0.582	0	0.453	0
*a*17:1 w9c	0.475	1.117	0.995	0	0.487	0.878	0.328	0.756		0	0	0.275	0.415	0	0	0	0
*i*17:0	1.299	2.798	2.466	3.966	1.260	1.952	0.797	1.697		0.406	0.399	0.645	0.965	0.693	0	0.649	0.314
*i*17:0	1.661	4.126	3.004	5.270	1.431	2.869	1.006	1.860		0.570	0.600	0.808	1.085	0.777	0.158	0.704	0.379
17:1 w8c	0.475	1.056	0.880	1.054	0.906	1.292	0.447	1.111		0.399	0.515	0.481	0.978	0.523	0	0.523	0.740
17:0 cyclo w7c	2.637	7.555	4.145	7.854	3.079	7.063	2.223	5.704		1.328	1.219	1.982	2.113	1.907	0.424	1.839	0.664
17:0	0.519	1.101	0.944	0	0.333	0.772	0	0.615		0	0	0.214	0.333	0	0	0	0
10-Me 17:1 w7c	1.551	1.080	1.942	1.456	1.391	0.831	1.170	1.562		0.631	0.322	0.755	0.469	1.566	0.354	1.592	0.972
16:0 2OH	0	0.315	0	0.545	0	0.246	0	0	0	0	0	0	0	0	0	0	0
10-Me 17:0	0.433	2.018	0.906	1.185	0.440	1.081	0.254	0.620		0.305	0	0.337	0.354	0.352	0	0.280	0
18:3 w6c	0.836	2.177	0.948	1.976	1.272	1.244	1.005	1.205		0.505	0.714	0.582	0.810	0.518	0.428	0.559	0.672
*i*18:0	0	0	0	1.639	0	0.951	0	0	0	0	0	0	0	0	0	0	0
18:2 w6c	1.329	5.760	2.871	5.557	1.232	2.373	0.847	1.442		0.261	0.268	0.688	1.881	0.518	0	0.746	0.579
18:1 w9c	4.705	17.411	10.818	37.331	5.007	10.718	2.986	7.235		1.440	1.642	2.062	4.289	2.973	0.368	2.543	2.018
18:1 w7c	10.217	35.237	21.833	49.740	8.963	26.956	5.488	17.423		2.429	2.938	3.891	6.378	4.518	0.473	4.582	1.979
18:1 w5c	1.480	3.900	3.106	8.992	0.862	1.512	0.522	1.675		0	0	0.331	0.532	0.562	0	0.457	0
18:0	2.485	3.071	3.572	6.822	2.692	3.758	1.835	2.713		0	0.238	0.174	0.557	0.441	0	0.584	0
10-Me 18:1 w7c	0.759	2.392	1.546	5.151	0.483	1.733	0.313	1.032		0	0	0.190	0.372	0.219	0	0.224	0
3OH 17:0	0.457	0.435	0.498	0.415	0.470	0.338	0.317	0.405		0.219	0.228	0.317	0.250	0.320	0	0.260	0
10-Me 18:0	1.624	4.265	3.346	5.033	1.200	2.743	0.876	1.966		0.386	0.406	0.632	0.849	0.895	0	0.591	0.556
19:3 w6c	0.142	0.263	0.223	0.531	0.264	0.259	0.134	0.167		0.077	0.109	0.077	0.097	0.076	0.125	0.185	0.163
19:1 w8c	0	0.364	0.275	2.365	0	0	0	0.237		0	0	0	0	0	0	0	0
*cy*19:0 w7c	4.225	16.040	9.559	54.931	2.291	7.064	1.732	3.983		0.359	0.257	0.598	0.865	0.568	0	0.598	0.081
20:4 w6c	0	1.434	0.849	0.829	0.381	0.645	0.213	0.775		0	0	0	0.259	0	0	0.520	0

20:5 w3c	0	0.725	0.215	0.555	0.186	0.249	0.000	0.411	0	0	0.145	0	0	0	0.388	0
20:2 w6c	0	1.478	1.208	2.780	0	0	0	0	0	0	0	0	0	0	0	0
20:1 w9c	0	0	0	0	0.329	0	0.230	0	0	0	0.191	0.306	0.240	0	0	0
20:0	0	0.491	0.397	1.141	0.202	0.398	0.000	0.286	0	0	0	0	0	0	0	0
10-Me 20:0	1.032	0.951	0.945	3.655	3.243	3.590	0.109	1.286	0.491	0.333	0.651	0.576	0.720	0.508	0.725	1.195
21:1 w3c	0	0.342	0.468	0	0.197	0.185	0.184	0.662	0	0	0.172	0	0.244	0	0.233	0
22:1 w8c	0	0	0	0.541	0.359	0.438	0	0	0	0	0	0	0	0	0	0
22:0	0	0.244	0	1.146	0	0.297	0	0	0	0	0	0	0	0	0	0
24:0	0	0.344	0	1.588	0.222	0.553	0	0	0	0	0	0	0	0	0	0

Data are expressed in nmol g dry soil^{-1}

i = iso-, a = aniso- refers to the group with *iso* or *ante-iso* methyl branching.

10-Me = 10-methyl.

cy = cyclo

Supplementary Information

Table S2. Plant community composition in the four summits and aspects at "Ordesa and Monte Perdido" National Park (Central Pyrenees).

Plant community composition		Summits Acuta (2,232 m)				Custodia (2,519 m)				Tobacor (2,769 m)				Olas (3,022 m)			
		N	S	E	W	N	S	E	W	N	S	E	W	N	S	E	W
Achillea millefolium L.	*Achi*	0	3	0	1	0	0	0	0	0	0	0	0	0	0	0	0
Agrostis capillaris L. subsp. *capillaris*	*Agro*	0	1	0	0	0	0	0	0	0	0	0	0	0	0	0	0
Alchemilla alpina L. subsp. *saxatilis* (Buser) Rouy & Camus	*Alpi*	1	1	0	0	0	1	0	1	0	0	0	0	0	0	0	0
Alchemilla catalaunica Rothm.	*Acata*	1	0	2	0	0	0	0	0	0	0	0	0	0	0	0	0
Androsace ciliata DC.	*Andro*	0	0	0	0	0	0	0	0	0	0	0	0	3	1	1	1
Androsace villosa L.	*Avill*	2	0	0	0	0	0	0	0	0	0	0	0	0	0	0	0
Androsace vitaliana (L.) Lapeyr. *subsp. vitaliana*	*Avita*	2	0	0	0	0	1	0	0	0	0	0	0	0	0	0	0
Antennaria dioica (L.) Gaertn.	*Anten*	1	0	1	0	0	0	0	0	0	0	0	0	0	0	0	0
Anthyllis gr. *vulneraria* L.	*Anthy*	0	0	1	0	0	1	0	1	0	0	0	0	0	0	0	0
Arenaria moehringioides J. Murr	*Arena*	0	0	0	0	0	1	0	0	0	0	0	0	0	0	0	0
Arenaria purpurascens Ramond ex DC.	*Apur*	0	0	3	0	2	1	2	3	1	1	0	2	0	0	0	0
Aster alpinus L.	*Aster*	2	1	1	0	0	0	0	0	0	0	0	0	0	0	0	0
Borderea pyrenaica Bub. & Bordère ex Mieg.	*Borde*	2	0	2	0	0	0	0	0	0	0	0	0	0	0	0	0
Botrychium lunaria (L.) Sw.	*Botry*	0	0	0	0	0	0	1	0	0	0	0	0	0	0	0	0

Bupleurum ranunculoides L.	*Buple*	2	0	2	0	0	0	0	0	0	0	0	0	0	0	0	0
Calluna vulgaris (L.) Hull	*Callu*	0	0	0	1	0	0	0	0	0	0	0	0	0	0	0	0
Campanula cochlearifolia	*Campa*	0	0	0	0	1	0	0	0	0	0	0	0	0	0	0	0
Campanula scheuchzeri Vill.	*Csche*	1	1	2	2	0	0	0	0	0	0	0	0	0	0	0	0
Carduus carlinifolius Lam. subsp. *carlinifolius*	*Cardu*	0	2	1	0	0	0	0	0	0	0	0	0	0	0	0	0
Carduus carlinoides Gouan	*Ccarli*	0	0	0	0	0	0	0	0	0	1	1	0	0	0	0	0
Carex caryophyllea Latourr.	*Carex*	0	0	0	2	0	0	0	0	0	0	0	0	0	0	0	0
Carex ornithopoda Willd.	*Corni*	1	0	2	0	0	1	0	0	0	0	0	0	0	0	0	0
Carex parviflora Host	*Cparvi*	0	0	0	0	0	0	0	0	0	0	0	1	0	0	0	0
Carex rupestris All.	*Crupes*	1	0	2	0	0	0	0	0	0	0	0	0	0	0	0	0
Cerastium alpinum L.	*Ceral*	0	0	0	0	0	0	0	0	0	0	0	0	1	0	0	0
Cerastium arvense L.	*Cerarv*	0	1	0	0	0	2	0	0	0	0	0	0	0	0	0	0
Crepis pygmaea L.	*Crepi*	0	0	0	0	0	0	0	0	0	1	1	1	0	1	0	0
Dianthus benearnensis Loret	*Dian*	0	1	0	1	0	0	0	0	0	0	0	0	0	0	0	0
Erigeron cf. *alpinus* L.	*Erige*	2	0	1	1	0	2	0	1	0	0	0	0	0	0	0	0
Eryngium bourgatii Gouan	*Eryng*	0	1	0	0	0	0	0	0	0	0	0	0	0	0	0	0
Euphrasia alpina Lam. subsp. *alpina*	*Euphr*	0	1	0	0	0	0	0	0	0	0	0	0	0	0	0	0
Euphrasia salisburgensis Funck	*Esalis*	0	0	1	1	0	1	0	0	0	0	0	0	0	0	0	0
Festuca alpina Suter subsp. *riverae* Chas, Kerguélen & Plonka	*Falpi*	0	0	0	0	0	0	0	0	0	0	0	0	1	0	0	0

Festuca eskia Ramond ex DC.	*Feskia*	0	3	0	4	0	0	0	0	0	0	0	0	0	0	0	0
Festuca gautieri (Hack.) K. Richt. subsp. *scoparia* (A.Kerner & Hack.) Kerguélen	*Fgaut*	4	5	3	0	2	5	3	2	0	0	0	0	0	0	0	0
Festuca glacialis (Miég. Ex Hack.) K. Richt.	*Fglaci*	0	0	0	0	0	1	1	1	0	0	1	1	0	0	0	0
Festuca pyrenaica Reut.	*Fpyre*	0	0	0	0	1	0	2	1	0	0	1	0	0	0	0	0
Festuca gr. *rubra* L.	*Frubra*	0	0	0	1	0	0	0	0	0	0	0	0	0	0	0	0
Galium marchandii Roem. & Schult.	*Gmarc*	0	1	0	1	0	0	0	0	0	0	0	0	0	0	0	0
Galium pyrenaicum Gouan	*Gpyren*	0	0	0	0	2	0	2	2	1	1	2	2	0	0	0	0
Gentiana nivalis L.	*Gentia*	0	0	0	0	0	1	0	1	0	0	0	0	0	0	0	0
Gentiana verna L.	*Gverna*	0	0	0	0	0	1	0	1	0	0	0	0	0	0	0	0
Gentianella campestris (L.) Börner	*Gcamp*	1	0	0	0	0	0	0	0	0	0	0	0	0	0	0	0
Geranium cinereum Cav.	*Geran*	1	1	3	0	2	3	1	4	0	0	1	0	0	0	0	0
Helictotrichon sedenense (Clarion ex DC.) Holub	*Hsede*	2	0	2	0	3	3	2	3	0	1	1	0	0	0	0	0
Hippocrepis comosa L.	*Hippo*	0	1	0	0	0	0	0	0	0	0	0	0	0	0	0	0
Jasione laevis Lam. subsp. *laevis*	*Jasion*	1	0	2	0	0	0	0	0	0	0	0	0	0	0	0	0
Juniperus communis L. subsp. *hemisphaerica* (C. Presl.) Nyman	*Junip*	0	0	0	1	0	0	0	0	0	0	0	0	0	0	0	0
Leontodon hispidus L. subsp. *hispidus*	*Leon*	1	1	1	0	0	0	0	0	0	0	0	0	0	0	0	0
Leontodon pyrenaicus Gouan	*Lpyren*	0	0	0	1	1	0	0	1	0	0	0	0	0	0	0	0
Leontopodium alpinum Cass.	*Lalpin*	1	0	0	0	0	0	0	0	0	0	0	0	0	0	0	0
Leucanthemopsis alpina (L.) Heywood	*Leucan*	0	0	0	0	0	1	1	1	1	1	1	1	0	0	0	0

Linaria alpina (L.) Mill.	*Linar*	0	0	0	0	0	0	1	0	0	1	0	1	0	1	1	1
Lotus corniculatus L.	*Lotus*	0	1	1	0	0	2	1	3	0	0	0	0	0	0	0	0
Luzula spicata (L.) DC:	*Luzu*	0	0	0	0	0	1	0	0	0	0	0	0	0	0	0	0
Minuartia cerastiifolia (Ramond ex DC.) Graebn.	*Minu*	0	0	0	0	0	0	0	0	0	0	0	0	0	1	1	1
Minuartia verna (L.) Hiern	*Miver*	2	1	1	0	0	0	0	0	0	0	0	0	0	0	0	0
Myosotis alpestris F.W. Schmidt	*Myoso*	0	0	0	0	0	1	0	0	0	0	0	0	0	0	0	0
Nardus stricta L.	*Nardus*	0	0	0	2	0	0	0	0	0	0	0	0	0	0	0	0
Oxytropis neglecta Gay ex Ten.	*Oxytro*	0	0	0	0	1	0	0	1	0	1	0	0	0	0	0	0
Phyteuma hemisphaericum L.	*Phyteu*	0	1	0	0	0	0	0	0	0	0	0	0	0	0	0	0
Phyteuma cf. orbiculare L.	*Porbi*	1	0	2	0	0	0	0	0	0	0	0	0	0	0	0	0
Pilosella lactucella (Wallr.) P. D. Sell	*Pilos*	0	1	1	0	0	2	1	0	0	0	0	0	0	0	0	0
Pilosella officinarum F.W. Schultz	*Poffi*	0	0	0	2	0	0	0	0	0	0	0	0	0	0	0	0
Pimpinella saxifraga	*Pimpi*	0	0	1	0	0	0	0	0	0	0	0	0	0	0	0	0
Plantago alpina L.	*Palpin*	0	2	0	0	0	0	0	0	0	0	0	0	0	0	0	0
Plantago monosperma Pourr. subsp. *monosperma*	*Pmono*	1	1	0	0	0	0	0	0	0	0	0	0	0	0	0	0
Poa alpina L.	*Poalp*	0	2	0	0	2	4	1	4	0	0	1	1	1	1	1	0
Polygala alpestris Rchb.	*Poly*	0	0	0	0	0	1	0	0	0	0	0	0	0	0	0	0
Potentilla neumanniana Rchb.	*Poten*	1	2	1	0	0	2	0	1	0	0	0	1	0	0	0	0
Potentilla nivalis Lapeyr.	*Pniva*	0	0	0	0	0	0	0	0	0	0	0	0	0	1	0	0
Pritzelago alpina (L.) Kuntze	*Pritz*	0	0	0	0	0	0	0	0	0	0	0	1	1	0	1	1

Ranunculus gouani Willd.	*Ranun*	0	0	0	0	0	1	0	0	0	0	0	0	0	0	0	0
Ranunculus parnassifolius L. *subsp. heterocarpus* Küpfer	*Rparna*	0	0	0	0	1	0	1	0	0	0	0	0	0	0	0	0
Rhinanthus pumilis (Sterneck) Pau subsp. *pumilus*	*Rhina*	1	1	0	0	0	0	0	0	0	0	0	0	0	0	0	0
Saponaria caespitosa DC.	*Sapon*	0	1	0	0	0	0	0	0	0	0	0	0	0	0	0	0
Saxifraga aizoides L.	*Saxai*	0	0	0	0	1	0	1	0	0	0	0	0	0	0	0	0
Saxifraga pubescens Pourr. subsp. *iratiana* (F.W. Schulz) Engl. & Irmscher	*Sapu*	*0*	0	0	0	0	0	0	0	0	0	0	0	2	0	1	0
Saxifraga moschata Wulfen	*Samos*	0	0	0	0	0	1	1	1	0	0	2	0	0	0	0	0
Saxifraga oppositifolia L.	*Saxop*	0	0	0	0	1	0	2	1	1	0	0	1	2	1	1	0
Saxifraga paniculata Mill.	*Sapan*	1	0	0	0	0	0	0	0	0	0	0	0	0	0	0	0
Sedum atratum L.	*Sedum*	0	0	0	0	0	0	1	0	0	0	1	0	0	0	0	0
Sideritis hyssopifolia L.	*Sider*	0	1	0	0	0	0	0	0	0	0	0	0	0	0	0	0
Silene acaulis (L.) Jacq.	*Silen*	1	0	0	0	1	0	0	0	0	0	0	0	1	1	1	0
Silene ciliata Pourr.	*Sicil*	0	1	1	0	0	0	0	0	0	0	0	0	0	0	0	0
Solidago virgaurea L.	*Solid*	0	1	1	1	0	0	0	0	0	0	0	0	0	0	0	0
Taraxacum sp.	*Tarax*	0	1	1	0	0	1	0	0	0	0	0	0	0	0	0	0
Thymelaea tinctoria (Pourr.) Endl. subsp. *nivalis* (Ramond) Nyman	*Thyme*	0	1	0	0	0	0	0	0	0	0	0	0	0	0	0	0
Thymus praecox Opiz	*Thymus*	2	1	2	1	1	1	1	2	0	1	0	0	0	0	0	0
Trifolium alpinum L.	*Trifol*	0	0	0	5	0	0	0	0	0	0	0	0	0	0	0	0
Trifolium pratense L.	*Tripra*	0	2	2	0	0	2	0	0	0	0	0	0	0	0	0	0

Trifolium thalii Vill.	*Tritha*	0	0	0	0	0	2	0	3	0	0	0	1	0	0	0	0
Trifolium repens L.	*Trire*	0	1	0	0	0	0	0	0	0	0	0	0	0	0	0	0
Veronica nummularia Gouan	*Vernu*	0	0	0	0	0	1	0	1	0	0	0	1	0	0	0	0
Veronica officinalis L.	*Veron*	0	1	0	0	0	0	0	0	0	0	0	0	0	0	0	0

Numbers indicate the abundance/dominance index used (from Braun-Blanquet cover-abundance scale method) and refers to the percentage of the area covered: 5 (>80%), 4 (61-80%), 3 (41-60%), 2 (21-40%), 1 (11-20%), + indicate less than 6%.

Braun-Blanquet, J. 1932. Plant sociology (Transl. G. D. Fuller and H. S. Conrad). McGraw-Hill, New York. 539 pp.
